# Unpacking the ethics of using AI in primary and secondary education: a systematic literature review

**DOI:** 10.1007/s43681-025-00770-0

**Published:** 2025-06-23

**Authors:** Michał Wieczorek, Mohammad Hosseini, Bert Gordijn

**Affiliations:** 1 Dublin City University, Dublin, Ireland; 2 Northwestern University, Evanston, United States

**Keywords:** Artificial intelligence, Education, Ethics, Primary and secondary schools, Systematic literature review

## Abstract

This paper provides a systematic review of the literature discussing the ethics of using artificial intelligence in primary and secondary education (AIPSED). Although recent advances in AI have led to increased interest in its use in education, discussions about the ethical implications of this new development are dispersed. Our literature review consolidates discussions that occurred in different epistemic communities interested in AIPSED and offers an ethical analysis of the debate. The review followed the PRISMA-Ethics guidelines and included 48 sources published between 2016 and 2023. Using a thematic approach, we subsumed the ethical implications of AIPSED under seventeen categories, with four outlining potential positive developments and thirteen identifying perceived negative consequences. We argue that empirical research and in-depth engagement with ethical theory and philosophy of education is needed to adequately assess the challenges introduced by AIPSED.

## 1 Introduction

This systematic literature review answers the following research question: What ethical implications of the use of AI in primary and secondary education have been identified in the literature?

The use of AI in primary and secondary education (AIPSED) has made headway in many countries and has been widely debated. Developments in machine learning (ML) and large language models (LLMs) could increase the efficacy of AIPSED, while also offering new opportunities for creating personalized content for individual learners. Given the quick uptake of AIPSED and its perceived potential to improve education, it is prudent to synthesise the scholarly debate in a systematic review focused on the ethical implications of AIPSED. A pilot search conducted on 6 September 2023 (using Scopus, Web of Science and Google Scholar and a combination of the following terms: “AIED”, “AI in education”, “artificial intelligence in education”, “review” and “ethic*”) revealed no previous systematic reviews of literature on the ethics of AIPSED. We were able to identify 5 related reviews, but none of them fully responded to our research question as they either focused only on a subset of AIED tools or briefly discussed a limited subset of ethical implications (often as part of a wider study):

1. Lameras and Arnab’s review ^[1(p. 1)]^ briefly mentioned the ethical aspects of AIED, but the main question of their study was “What do we mean by Artificial Intelligence in Education?”
2. Zawacki-Richter et al.’s review [2] focused on AI applications in higher education, and despite discussing some ethical concerns (e.g., privacy or inclusivity), it was not primarily concerned with ethics.
3. Yan et al.’s review [3] dealt exclusively with LLMs but only addressed ethical implications connected to privacy, equal access and beneficence.
4. Memarian & Doleck reviewed ^[4(p. 2)]^ “fairness, accountability, transparency and ethics” regarding AI use in higher education, but mostly focused on how these principles are defined, teaching of AI ethics or making AI itself more ethical– neither of which falls within the scope of our review.
5. Crompton et al. reviewed [5] the challenges surrounding AI use in K-12, but they discussed ethical concerns (e.g., privacy or bias) alongside practical obstacles for the integration of AI in schools.

Furthermore, while we were working on this review, Mouta et al. [6] published a related review to ours, adopting quantitative and bibliometric methods and focusing only on certain implications of the ethics of AIPSED (e.g., capacity building, cultural diversity, ethics of emotion recognition).

In our review, we adopt a broad understanding of AI in accordance with UNICEF’s definition: “AI refers to machine-based systems that can, given a set of human-defined objectives, make predictions, recommendations, or decisions that influence real or virtual environments. AI systems interact with us and act on our environment, either directly or indirectly. Often, they appear to operate autonomously, and can adapt their behaviour by learning about the context”^1^. This broad definition covers a wide variety of existing AI tools, particularly ML algorithms, LLMs and expert systems, which, arguably, are the most common AI systems used in education [7].

We have four reasons for focusing on primary and secondary education: (1) They are mandatory in most countries, and changes introduced by AI will have far-reaching impacts; (2) They involves minors, and potential negative implications of AI and harms are particularly concerning; (3) Since local/national bodies (e.g., ministry of education) determine policies, curricula and methods used, teachers have much less influence than, higher education lecturers about whether/how AI should be used. (4) Academics often focus on AI use in higher education, perhaps because it is a familiar context. As such, the ethical implications pertaining to AIPSED remain underexplored.

Consequently, we will *not* consider impacts that are *exclusive* to higher education (e.g., on academic freedom) or analyses that refer *only* to higher education.^2^ Our review followed the PRISMA-Ethics guidelines [8] and the protocol was registered on OSF (anonymised for peer review).

## 2 Method

### 2.1 Eligibility criteria

We included sources that:

1. Focus on AIPSED.
2. Discuss the ethical implications of AIPSED.
3. Are peer reviewed (e.g., journal articles, book chapters or books).^3^
4. Are written in English.

### 2.2 Databases used

We used the following databases to search the literature:

(1) Scopus.
(2) Web of Science.
(3) Academic Search Complete.
(4) Google Scholar.

### 2.3 Search terms

We combined two sets of search terms– one containing terms referring to AIPSED and one covering ethical terms. The full list of terms and the used search strings can be found in Supplementary File 1.

### 2.4 Search and selection of sources

M.W. searched all four databases on 9 October 2023, retrieving 627 documents. In the case of edited volumes, each of the chapters was separately considered, which resulted in finding nine additional chapters. The exclusion of duplicates (with Covidence and manually) left us with 461 sources. M.W. screened the title, abstract and keywords, assessing them for full-text review according to our inclusion criteria (if abstracts were unavailable, introductions were reviewed instead). M.H. and B.G. verified that the selected sources met the eligibility criteria by independently screening the title, abstract and keywords of 25 randomly selected sources. There were no disagreements between the authors at this stage, and we consequently selected 102 sources for full-text reading.

**Fig. 1:**
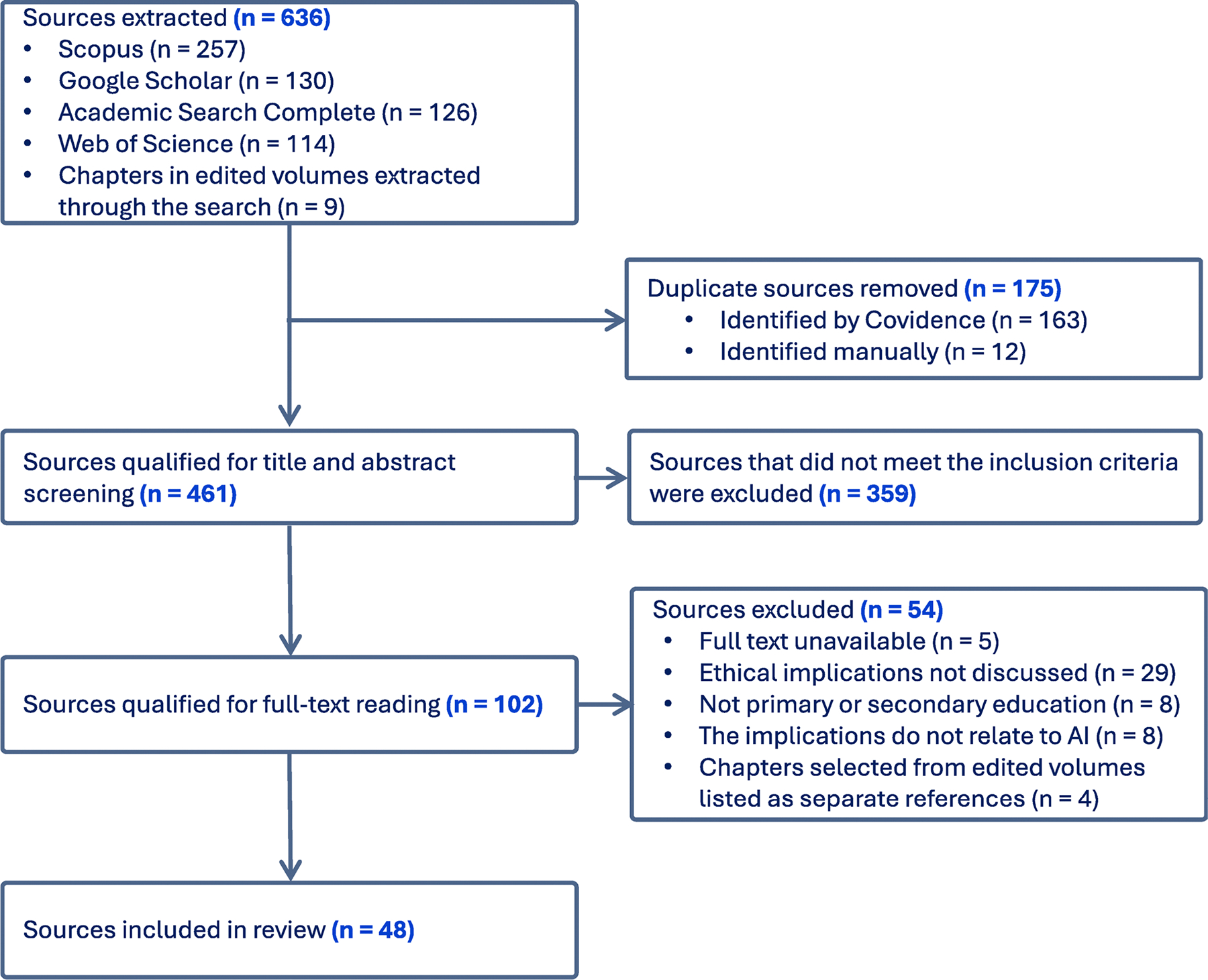
PRISMA Flowchart

M.W. read the sources to determine their elgibility. M.H. and B.G. each read 10 randomly-selected sources and independently checked their eligibity. There was no disagreements and we consequently included 48 sources in the review (see Supplementary File 2). A breakdown of the selection process, as well as the reasons for exclusion are available in the PRISMA flowchart (Fig. 1).

## 3 Data extraction and analysis

We adopt a broad understanding of ethical implications that is common in applied ethics reviews [10, 11]. For example, an ethical analysis of a new medical intervention would typically consider aspects like benefits to patients and potential harms, but also the impacts on medical professionals and medical practices. Adopting a similar approach to the ethics of AIPSED will allow the readers to better understand the balancing act between the advantages and drawbacks of AIPSED. We consider this especially pertinent as many analyses in AI ethics focus only on the negative impacts or on a narrow range of issues (e.g., fairness, accountability, transparency, privacy) and do not offer the full picture of the ethical implications of AI use.

To analyze the selected corpus, M.W. analyzed the included sources using a qualitative and inductive thematic approach [12]. The full text of the sources (rather than specific sections) were analyzed and individual arguments and issues were coded with corresponding ethical issues. Codes were either based on the sources’ own identification of ethical aspects related to the use of AIPSED tools, or on our ethical expertise, particularly when ethical concerns were implied but not explicitly labeled or framed in conventional ethical terms. We deliberately decided *not* to rely on a checklist or other prior identification of potential ethical implications to avoid influencing our ethical convictions or the adopted perspective. We accounted for, and gave equal weight to all implications identified in the sources, as is customary in literature reviews in ethics [10, 13]. After the initial coding, specific implications were grouped into themes to improve clarity and identify commonalities. For this purpose, each source was mapped in a Microsoft Excel spreadsheet and the identified arguments were listed in separate columns to allow for cross-comparisons and to report how many times each theme occurs in the sample (see Table 1). The process led to the identification of 17 themes, which were later independently reviewed and approved by M.H. and B.G. Four themes deal with the perceived benefits of AIPSED, while 13 outline potential concerns (see Table 1).

### 3.1 Quality appraisal

Since presenting a full synthesis of the debate was our goal, when coding, the arguments were given equal weight and we did not attempt to examine their validity. However, we reflected upon the overall state of the literature in the Discussion section. Moreover, we also distinguished the depth with which authors engage a given implication. In the results section, we first summarise the most common and generic claims about each of the ethical implications of AIPSED and then outline the more detailed analyses present in the literature.

## 4 Results

### 4.1 Benefits of using AIPSED

#### 4.1.1 Facilitating teachers’ jobs

This theme collects the discussion of AIPSED’s positive impact on teachers’ tasks. AIPSED enables automation of tedious or time-consuming teaching tasks (e.g., assessment and administration), thus leaving educators more time to focus on students, or increasing overall productivity [1, 3, 14–27]. AIPSED could also help teachers prepare materials and lesson plans [22, 28], and when this happens in real time, it enables dynamic teaching [15].

Some note AIPSED’s capabilities to analyse performance and behaviour, which can help teachers identify how well students understand the material, and thus, manage the classroom better by adapting their practices and time-management to individual learners’ needs [1, 17, 18, 29–33]. Moreover, AIPSED leads to greater student engagement, it reduces the time and effort required to deal with classroom management and allow the educators to focus on teaching [34]. AIPSED can also be helpful in orchestrating the increasing number of various resources deployed in schools [30].

In terms of assessment, AIPSED would limit the impact of human subjectivity in decisions about students’ achievements [19] and assess without the influence of irrelevant factors, such as students’ backgrounds [32]. Similarly, fair AI would not have favourite students or would not be guided by racist stereotypes [15]. Although AIPSED exhibits biases, it might still be an improvement if it could assess students more objectively than an *average* teacher and students might prefer to be assessed by AIPSED [35]. It is possible for AIPSED to challenge the decisions and presumptions of biased human teachers (e.g., by supplying data incompatible with those decisions and beliefs) and thus gradually reduce their bias [36].

Four sources discuss upskilling and empowering effects that AIPSED can have on teachers [1, 22, 24, 33]. Su & Yang claim that the use of AIPSED will help teachers improve their teaching practices, but do not provide further reasoning for this statement [33]. Lameras & Arnab argue that such improvements will occur as the introduction of AIPSED will help teachers make better use of students’ data and adapt their practices accordingly [1]. Similarly, Mohammed and Watson note that teachers might reflect on the data supplied by AIPSED and use it to improve their teaching practices [1]. In turn, Kasneci et al. claim that LLMs will keep teachers up to date with new methodologies and teaching materials by providing digestible summaries and explanations [22].

#### 4.1.2 Reducing inequalities

Under this theme, we discuss AIPSED’s contribution to alleviating inequalities in access to education and educational achievement. Many authors discuss the potential of AIPSED for improving adaptability, inclusion and access to education, which might reduce educational inequalities (although some list this as a general feature of AIPSED instead of attributing it to specific functions [37, 38]). In particular, authors note that personalisation techniques may tailor content to the needs and circumstances of individual students, which will help close educational attainment gaps [14–17, 25–28, 39, 40]. AIPSED could also adapt the rate of learning to a student’s progress or fit the content to their preferred learning methods [18].

Such techniques are considered beneficial for students with special educational needs connected to disabilities, learning difficulties and neurodiversity [15, 17, 18, 22, 26, 34, 39, 41]. Some students with autism respond well to, and seem more comfortable with, AI than with human caretakers and educators [24] while speech recognition techniques support students with hearing difficulties, e.g., by helping them distinguish between ambient noise and relevant information [19].

AIPSED is also believed to improve access to education (Schiff and Nemorin et al. mention increased access but do not attribute it to specific factors [41, 42], while Smuha notes the ability of AIPSED to scale-up education without connecting it to access [40]). This can happen when AIPSED facilitates distance learning for students living in remote areas or otherwise unable to attend school [17, 18, 27, 28] or provides personalised tutoring or high quality education to students from low-income families who otherwise could not afford it [18, 43]. In particular, AIPSED could be beneficial to developing and low-income countries impacted by teacher shortages, as well as a lack of resources and crucial infrastructure such as school buildings [18, 25, 31]. Yan et al. suggest that the increased development of non-English AIPSED is a particularly welcome trend that improves education around the globe [3].

The use of AIPSED could reduce the ability of better-off parents and students to game the system of school admissions, because AI is less susceptible to tactics that prove successful in gaining approval of human teachers and administrators [20]. AIPSED can also prove helpful for smaller and less digitised schools and school systems without significant student data as it would allow them to extrapolate predictions and recommendations based on other schools’ data, thus contributing to data exchange and greater equity between different educational institutions [20].

#### 4.1.3 Improving educational outcomes

This section synthesises the discussion of AIED improving students’ grades and school performance, as well as their acquisition of new skills with the help of the technology. Several authors mention that the use of AIPSED leads to improved educational outcomes like higher test scores [14, 20], and the arguments typically attribute this to two factors. One is AIPSED’s ability to adapt content and available resources to particular students’ needs and abilities [17, 18, 21, 24, 27, 28, 32, 33, 39, 42]. Another relates to the increased engagement attributed to AIPSED-assisted learning [18, 22, 24, 33]. In this context ongoing developments in emotion recognition and corresponding techniques to elicit positive emotions in students may be relevant [29]. Du Boulay provides an example of an AI tool that would (falsely) convince students about dealing with a challenging task since associated excitement can be attributed to increased learning gains [30]. Alshahrani argues that increased engagement may be connected to interactive and conversational features of AIPSED, which demand greater student participation [28] and Du Boulay discusses a conversational agent that would deliberately induce confusion or moderate a discussion to increase student participation [30]. Reiss suggests that the use of AIPSED could increase students’ motivation [34].

AIPSED can also benefit students’ skills, knowledge and learning ability, although some authors merely list such possibilities in passing [3, 21, 24, 42, 44]. Many authors who engage in more depth with such claims focus on specific skills, for example noting that AI’s better performance in tasks like pattern recognition could support teaching reading and writing even if there are reasons to be sceptical about AI’s overall teaching ability to teach as a whole [45], or claiming that suggestions from tools like ChatGPT could improve students’ academic writing [33]. LLMs can improve writing ability by correcting grammatical and syntactic errors or helping students master specific writing styles (e.g., physics or literature), while students’ reading comprehension could be increased thanks to the ability of such tools to summarise and explain difficult texts [22]. AI’s affinity for teaching problem-solving and critical thinking skills is also relevant [22, 37], and there is research demonstrating AI’s positive impact on students’ interpersonal and intrapersonal skills (e.g., time management and self-regulation) [37].

When exploring AIPSED’s impact on learning and learning ability, Saputra et al. claim that the provision of novel concepts and nuancing existing material can help students learn more effectively, while also increasing their confidence, especially in the digital sphere [32]. Schiff argues that recognition of students’ emotions, engagement and personal circumstances could provide them with better cognitive and emotional support, thus allowing them to learn better [25]. Referring to this argument, Butt et al. claim that individual tutoring systems help students learn at least as efficiently as with the help of human tutors [18].

Finally, four texts highlight AIPSED’s potential to reduce drop out rates by identifying students who are at risk of failing their classes (e.g., due to learning difficulties) and suggesting interventions [14, 18–20], with similar methods applicable to fight absenteeism [20].

#### 4.1.4 Other benefits

Here, we collect arguments that do not neatly fit into any of the three previous categories of benefits, and which do not appear in the sources frequently enough to warrant their own analytical theme. Various papers discuss potential benefits that do not fit neatly into the former three themes. AI has potential to offer coaching, counselling or administrative support [18, 46], ability to analyse student data and suggest career pathways or anticipate students’ future trajectories [46]. It can also guide students and parents through administrative processes, such as registration or applying for financial aid [14, 20]. AIPSED might eventually analyse students’ emotional data to support them in attaining educational goals that go “beyond test scores and graduation” ^[25(p. 342)]^. The range of available tools and content could offer flexibility to students to choose subjects that fit their interests and career paths, while institutions would gain the ability to tailor technologies to their needs [25]. Moreover, AIPSED could increase the diversity of what is being taught by incorporating topics relevant to digital literacy, as well as moral and civic education [41]. However, it is unclear whether this is an opportunity inherent to AIPSED or merely a wish.

AI’s ability to monitor students has also been noted, as it could help identify instances of bullying [47], and it could help recognise fear, danger and behavioural problems, although there are concerns related to, e.g., discrimination [16]. Moreover, AIPSED could keep parents updated about their children’s progress and performance [17, 20].

AIPSED could reduce the environmental impact of education by making it less reliant on paper and in-person meetings (through blended learning), thus reducing the carbon footprint associated with commuting [28]. However, the source does not contrast these gains with the environmental impact of AIPSED.

### 4.2 Concerns about AIPSED

#### 4.2.1 Privacy challenges

The first concern pertains to how AIPSED can infringe on students’ and teacher’s privacy by harvesting their personal data and subjecting them to monitoring and surveillance, often without adequate consent. Most sources (36 out of 48) note that AIPSED compromises privacy of students and teachers [1, 5, 9, 14, 15, 17–20, 22, 23, 27, 32–34, 38, 39, 41, 42, 48]. Some highlight that even when data is anonymised, there is a potential for re-identification of students, especially when there are few students with particular characteristics (e.g., ethnicity, disability) in a given class or school [26, 49]. AIPSED would limit students’ ability to hide thoughts and emotions, while also greatly reducing their ability to keep some school-related information away from their parents [47]. There are uncertainties regarding students’ data in education, including what exactly counts as personal [29]. As such, there may be conflicts when one student wants to review the data relating to their interactions with peers, some of whom might have wished to have this data removed [50]. Moreover, while it is generally accepted that schools will collect students’ personal data (e.g., examination records), this is not the case for private entities supplying AIPSED [51]. Third-parties further complicate schools’ responsibility to protect students’ personal information, especially as AIPSED is not limited to educational data, because algorithms could also rely on other sources of information such as internet activity [21]. AIPSED could also use information obtained through illegal means, although the authors do not provide specific examples [52], and tools can be set up to collect students’ non-educational data, such as information about their home environment [24]. Authors also raise concerns that educational data could be later used for non-educational purposes against the will of students [3, 40, 44]. Furthermore, privacy regulations differ between countries and restrictions on data collection and use disincentivise development of AIPSED [31]. Regular privacy checkups should be organised at schools to assess potential risks and address some privacy challenges [45].

Others highlight consent for the use of AIPSED and the associated data processing [9, 14, 24, 27, 44, 50, 53, 54]. Some privacy policies are purposefully written in a confusing manner, so accepting them should not be considered consent [16]. Similarly, since privacy policies are wordy and difficult to understand, students might carelessly accept them to save time [21]. Smuha questions whether it is at all possible for students to consent to AIPSED considering that they are minors and schools wield disproportionate power compared to them [40]. Furthermore, she wonders whether teachers can give meaningful consent when AIPSED is adopted by their employers.

Some authors worry about limited possibilities for opting out of data collection and retention [9, 21, 50, 55], especially as minors cannot always make such decisions [53]. The majority might also pressure outliers to contribute their data to reap the collective benefits of AIPSED, or the costs and effort required to implement AIPSED might effectively not allow for opting out once the system is deployed [40].

Inadequate security and unauthorised access to data are believed to further complicate privacy in AIPSED [9, 18, 21–24, 27, 39, 40, 44, 53]. In this context, there are worries that students’ and teachers’ data may be targeted by cybercriminals [17, 48], or that the aggregation of student data risks attracting attention of malicious actors [38]. Authors also argue that the management of personal data is challenging and risk-laden [9, 23, 27, 31], while worrying about the burden data management would place on students and teachers [23, 29, 53]. Some authors claim that AIPSED might necessitate the development of new data stewardship practices and policies [18, 19].

Additional concerns revolve around data collection being used as a means of surveillance [9, 14, 15, 20, 21, 41–44, 48, 51–53, 55]. This could predominantly affect marginalised students who are already placed under more scrutiny in educational settings [16, 37, 45, 56], although teachers might also be excessively monitored [34, 56].

#### 4.2.2 Unfairness and low accuracy

This theme synthesises the discussion of how and why AIPSED algorithms can lead to unfair, biased and inaccurate outcomes/decisions. Concerns about algorithmic fairness and bias (and the resulting discrimination) are raised by many authors [1, 5, 9, 14, 17, 19, 21, 22, 27, 32, 33, 39, 41, 43–45, 48, 50–53, 55]. Specific concerns include a lack of attention to fairness in the development of AIPSED [3], excessive emphasis on certain pedagogies and learning styles [25, 36, 38], prioritising developers’ own cultures and worldviews [24, 25], presenting students with tasks poorly related with their ability that impede learning [29], difficulties surrounding the ongoing monitoring of the tools for bias [37], replication of historical biases present in educational practices and the society as a whole and classification of students according to reductive categories (e.g., demographic) rather than their individual characteristics that might be better indicators of their ability [16, 26, 35, 36, 54].

Discussing bias, Selwyn concedes that humans also exhibit prejudices, but argues that achieving the level of fairness equivalent to humans does not justify adopting flawed tools [56]. In turn, Williamson et al. argue that biases cannot be addressed by computational approaches to fairness and design changes as wider social changes might be necessary to affect the social functioning of the technology and its interplay with the users [46]. Furthermore, Smuha argues that it is difficult to detect and remove bias as some discriminatory outcomes might arise due to the use of proxies rather than legally protected indicators such as race or gender [40].

Two sources offer a breakdown of different kinds of bias (e.g., deployment bias and measurement bias) [38, 46], with Williamson et al. claiming that commercial AIPSED tools are the most biased [46]. In turn, Holstein & Doroudi provide an overview of various kinds of biases specificially in AIPSED, for example by discussing models which do not adequately differentiate between students’ ability and thus do not account for difference in learning progress [36]. Baker and Hawn review different sources of algorithmic bias, concluding that so far, most of the attention has been devoted to racial, nationality and gender biases in AIPSED [49]. They propose focusing on factors such as native language and dialect, disability, urbanicity, parental economic background, and socioeconomic status. Moreover, they advocate for greater granularity in exploring bias (e.g., currently Asian-Americans are treated as one homogenous category), more consideration of intersectionality, and more attention to the as-of-yet unknown biases.

Authors commonly cited low availability, low quality or low representativeness of training data as a factor contributing to bias [1, 15, 17, 18, 26, 37–41, 43, 44], especially as some learners might not want to have their data collected and used to train AI [53]. Holstein & Doroudi explain that if most training data comes from a specific group (e.g., white students), algorithms might prioritise (through feedback-based ML) accuracy for the overrepresented cohort [36].

It is not clear what constitutive a representative sample of learners that would help ensure unbiased functioning of the algorithms [49]. Even accurate real-world representation might not guarantee adequate data on minorities– there might not be enough students from marginalised backgrounds in a given population (e.g., a school) to ensure reliability [49]. Linking data from different stages of a student’s life also constitutes a major challenge for AIPSED as not factoring for the changes which the students are undergoing risks the creation of narrow and reductive profiles that are unlikely to produce accurate results [50]. Mohammed & Watson are concerned about the cultural diversity of data [24]. Even if minorities are represented, the lack of sensitivity to variance in cultural expression of distinctive psychological and behavioural factors might lead to discriminatory outcomes (e.g., the emotions of autistic students have been misconstrued by algorithms measuring engagement). Additionally, much of the existing data that could be used to train AIPSED is of low quality and often incomplete, sometimes consisting mostly of students’ grades [35]. As such, he worries that developers will train the models not with useful, but with easy to gather information. Data for STEM subjects is more readily available, which might contribute to the priotization of these subjects over, e.g., philosophy or art [25].

Furthermore, authors argue that AIPSED can be inaccurate and erroneous [3, 5, 29, 33, 38], and fabricate information [15, 22]. Corbeil and Corbeil provide examples of a marking system failing students for submitting an assignment in a different format, or giving out grades altogether different than predicted by human teachers [19]. Leaton Gray argues that systems for monitoring engagement and behaviour need to operate on some assumptions about the students’ emotions and expressivity, but such assumptions are not necessarily correct [51]. Similarly, many AIPSED tools are not scientifically grounded, which will result in inaccuracies [40], while in-class assessment are inherently ambiguous and inexact, but AIPSED translates those to a rigid score [9]. Additionally, teachers are worried that they might be unable to verify information provided by AIPSED before it is used in the classroom [23]. and there are concerns about LLMs plagiarising copyrighted information [22].

Some authors are concerned about the overall reliability of information, content, assessment and recommendations provided by AIPSED [20, 24, 53], particularly due to the potential for false positives and false negatives [29, 45, 51] and stakeholders’ unsubstantiated belief in AIPSED’s credibility [51].

#### 4.2.3 Perpetuating injustice

In this section, we overview instances where AIPSED is seen as contributing to or introducing inequalities or otherwise characterised by authors as unjust. AIPSED can exacerbate educational inequalities, for example, when algorithms are inaccurate or less efficient for (marginalised) students underrepresented in the training data [9, 14, 20, 26, 33, 36–39, 41, 44–46, 49, 53], or when biased AIPSED entrenches and amplifies existing prejudices and patterns of exclusion [20, 26, 27, 35–38, 45, 46, 51, 53–56]. Moreover, socioeconomic disparities result in unequal access to digital technologies, making it more difficult for marginalised students to benefit from AIPSED [3, 9, 17, 18, 23, 36–38, 44, 46, 51, 52]. Other factors exacerbating inequalities included AIPSED’s lack of support for non-Western languages [3, 36], reliance on mainstream linguistic patterns and cultural references which may exclude non-native speakers and those who speak dialects [36, 38, 54, 56], inadequate support for learners with disabilities and special needs [36, 38, 46, 56], lack of input from marginalised communities regarding their needs and expectations [36, 56], validation of teachers’ discriminatory practices and beliefs [36], and enforcing behavioural patterns that exclude marginalised communities [54].

There are also concerns that some students might not engage with, or respond well to AIPSED and be left behind [9, 15, 19, 21, 26]. Some advantaged groups might benefit from AIPSED due to greater skills and resources, which would further increase the gap between the haves and have nots [9, 36, 38, 43, 53], thereby contributing to educational inequalities [56].

Such issues cannot be addressed by design interventions because inequalities intersect and are political in nature, requiring profound changes [16]. However, exacerbation of inequalities is a possibility in the early stage of AIPSED adoption, but wider adoption might counterbalance them [34].

Authors also observe that disparities of access to AIPSED also exist on the international level, for example due to differentiated access to digital tools and associated gaps in digital literacy in developing countries [17, 25, 48]. Moreover, AIPSED tools are primarily developed in, and targeting students in developed countries (particularly the USA), which might widen the global educational gaps [18, 24, 49], especially if the best tools are only available to, and usable by people from developed, primarily English speaking countries [3]. In particular, Schiff notes that AIPSED promotes Western-centric learning methods and values, which might render such technologies less effective or exclusionary when deployed in non-Western countries [25]. One respondent in the survey conducted by Holmes et al. warned of “cultural imperialism” when solutions designed for Western countries are deployed without being adapted for non-Western countries ^[9(p. 513)]^. Other authors echo similar concerns when they criticise cultural uniformity, and note that most features relevant to Western students will not work well for their non-Western counterparts [24, 31, 42], and the West-centric character of AIPSED can be seen as an aspect of contemporary colonialism [42]. Furthermore, Schiff worries that the adoption of off-the-shelf solutions in low-income countries will impede efforts to develop local educational infrastructure and confine them to use tools designed for a different context [25, 41].

Some authors mention the environmental impact of AIPSED and high energy cost [22, 40, 46, 56], and the technology’s reliance on rare minerals [56]. Nguyen et al. stress that those developing AIPSED should consider environmental sustainability and energy efficiency [44]. However, Selwyn argues that environmental concerns should make us question the use of AIPSED altogether [56].

### 4.3 Negative impact on autonomy and other harms

Under this theme, we collected arguments of AIPSED limiting students’ choices and freedom of thought, as well as negatively impacting their wellbeing. AIPSED could negatively impact students’ autonomy by influencing their behaviour through nudging and emotional manipulation [14–16, 23, 24, 26, 29, 33, 38, 41, 42, 44, 47, 48, 55]. For example, when students are exposed to a limited range of information [17, 40], when certain products, values and worldviews are (covertly) promoted [24, 40], or when students adapt their behaviour to be more easily recognisable by a system or because they feel watched [40, 45]. In this context, Schiff wonders whether all nudges should be disclosed to students and argues that while some influence might be beneficial, using manipulative techniques to achieve noble ends is problematic [25]. Nemorin et al., warn about harms to students’ dignity resulting from undue influence on their behaviour and freedom of thought [42]. Others are concerned that decisions made by AIPSED will restrict the choices available to students (regarding, e.g., which career to choose) [17, 29, 44, 53].

Some authors stress that children are vulnerable and impressionable and thus more susceptible to nudging and manipulation [9, 26, 40, 44]. This is particularly evident when young children interact with AI resembling humans, such as humanoid robots– they attribute them human-like characteristics and are thus more likely to trust them or consider them as friends [24]. There are also concerns about the negative impacts of AIPSED on autonomy because children are likely to be exposed to such tools over extended periods on a compulsory basis [38], and because students might be vulnerable to undue influences due to their age, but also their socioeconomic status or mental capacity [25].

There are also concerns about AIPSED harming students, although some authors note this possibility without providing examples [9, 21, 23, 30, 36, 44], or point to harmful consequences of inaccuracy, biases and discrimination [3, 14, 19, 20, 25, 36, 38, 40, 41, 46, 51, 53, 54, 56]. In turn, Selwyn worries about harms inflicted upon students who might have to misrepresent their gender when systems have been programmed to recognise only the male-female binary, as well as minority students being excessively monitored through AIPSED [56]. The latter concern is also raised by Madaio et al. who add that minority students are more vulnerable to disciplinary action resulting from such monitoring (e.g., the creation of a disciplinary record or encounters with the criminal justice system) [54]. Treviranus warns that since AI is particularly suited to teaching what it could automate, AIPSED might limit students’ future opportunities by making their skills more susceptible to automation [26].

AIPSED could also impact students’ mental health and well-being [40, 44] when it reduces the amount of social interactions among students [17], increases their stress levels [53] or reduces their sense of security [14] as a result of constant monitoring, and when students engage with poorly tested technologies unsuited to their age [23]. AIPSED could also reduce students’ confidence by highlighting their past failures as something to improve upon [47] or by contradicting their own perceptions (e.g., when a student is wrongly labelled as inattentive [30]). Li and Gu also note that students may get anxious about issues surrounding the system, such as its privacy implications [52].

AIPSED might result in students losing certain skills due to overreliance on technology or a lack of emphasis on, e.g., social interaction [17, 27, 39, 45, 53]. Reliance on automated monitoring and reduced interaction with teachers could reduce trust between students and educators [19, 37].

#### 4.3.1 Limitations of technology

In this section, we discuss implications arising from mismatches between AIPSED’s actual capabilities and the purposes towards which it is deployed. Since AIPSED algorithms are trained for narrowly defined tasks, they may not respond well to diverse educational situations [5, 9, 15, 18, 20, 24, 31, 33, 35, 40, 56, 57]. For example, authors point to shortcomings in recognising emotions or nuances of speech, such as irony [17–19, 25, 45, 51, 56]. While AI might be particularly suitable for teaching STEM subjects (as it is easier to model, e.g., math problems, computationally), this might lead to an excessive focus on STEM subjects in the curriculum and undervaluing humanities, social sciences and arts [18, 25, 34, 38]. Relatedly, authors claim that AIPSED focuses on a very limited set of values as evidenced by its endorsement of efficiency, productivity, cost-effectiveness and technical solutionism [26, 35, 46, 56].

Moreover, AIPSED systems primarily depend on quantitative data which by its very nature obscures context and relies on proxies and assumptions [53], and computational interpretations of the world are inherently reductive [42]. This makes it likely that relevant information will not be captured in and accounted for in AIPSED decisions. As such AIPSED may flag the risk of undesirable learning outcomes (e.g., a student failing a course), without accounting for the context which would explain *why* such outcomes are likely to occur [20, 35]. Similarly, human teachers are able to attribute different in-class occurrences to contextual factors (e.g., disruptive behaviour gets worse in bad weather), but this is not the case with AIPSED [23]. Madaio et al. see this as a significant limitation as AIPSED may help educators identify potential problems, but it is unable to offer solutions that would work in concrete situations [54], and it is problematic that AIPSED makes decisions without being able to identify their full impact [19]. However, Rowe claims that this should not be a problem as long as AIPSED is used only for a narrow subset of well-defined tasks that do not require general knowledge or reasoning skills [35]. Moreover, AIPSED tools do not always possess the capabilities they are purported to have, which further complicates their use [3, 56].

Given these limitations, Kasneci et al. are concerned that AIPSED might not adequately adapt to students’ needs [22], while others add that as children undergo significant changes throughout their education, it is likely that AI will struggle to keep up with these changes and will instead base its decisions on out-of-date student profiles [9, 50, 53]. Furthermore, some authors to question whether AI can actually automate certain tasks performed by teachers [17], especially as it is unable to navigate ethical dilemmas and value conflicts [35, 57], or model all of the social interactions in which teachers engage [24, 56]. Moreover, there are questions regarding which norms enacted by teachers AI should model [57], especially as sometimes teachers might be justified in tricking or manipulating their students (e.g., to make them argue for their positions), but it is not clear whether AI should be allowed to do so [29].

The need for the ongoing maintenance of AIPSED, especially due to the cost [3, 19, 22, 32, 33, 35, 46] and associated staff training needs are concerning [5, 18, 31, 46] (Butt et al., 2022; Crompton et al., 2022; Pinkwart et al., 2016; Williamson et al., 2023). AIPSED also requires constant monitoring on the part of the developers, e.g., to detect and address previously unnoticed biases [22, 37].

#### 4.3.2 Negative impact on learning

Here, we discuss the reasons for which AIPSED is seen as potentially reducing the quality and diversity of what and how students learn. There is little to no evidence concerning the effectiveness of AIPSED in improving learning, especially in the long term [20, 28, 33, 38, 42, 43, 45, 46, 50]. Since potential biases in AIPSED have not been studied, they cannot be counterbalanced in the development of new tools [49]. And even when evidence supporting the use of AIPSED is presented, it is unclear what aspect(s) of the systems contributed to its efficacy, thereby challenging further development [20]. There are also reasaons to criticise the promises surrounding personalised learning and– since no universally agreed upon definitions of personalisation exists, determining what developers mean with personalisation in education is challenging [46]. Due to the low quality of reporting standards, many studies on the effectiveness of new tools cannot be replicated, casting doubt on the validity of the findings and their impact on learning [3]. Crompton et al. observe that all sources on AIPSED they reviewed (*n* = 169) contained calls for more research on efficacy [5].

AIPSED may reduce the quality of education, reduce students’ performance or fail to respond to their needs [3, 9, 22]. AIPSED might negatively impact learning by distracting students or making them focus only on the engaging parts of a lesson [5, 17], while constant monitoring of students might lead to distrust and increased drop-out rates [37]. AIPSED might also divorce learning from real-life experience and make the content abstract [39], and its use might be accompanied by an increase in class sizes [25].

Some authors argue that pedagogies employed in AIPSED are not reflective of the existing evidence and best practices [9, 16, 39, 45, 52, 56]. AIPSED is especially prone to framing learning as passive acquisition of knowledge and placing little emphasis on other aspects of education such as social and character development [34, 35, 38, 46]. However, such criticisms may primarily be applicable to early developments as contemporary AIPSED tools provide students with more agency [43]. Madaio et al. argue that existing pedagogical practices are inherently unjust and AIPSED’s reliance on them risks entrenching inequalities and discrimination at schools [54].

Another concern relates to the students’ dependence of technology, with authors arguing that AIPSED might reduce independent thought [14] and problem-solving without devices [22, 45]. Similarly, AIPSED is seen as reducing engagement by impacting students’ motivation to explore topics outside of the algorithms’ suggestions [20] or encouraging laziness when students would rather use the help of AI than work and seek information on their own [22, 40].

Automation brought by AIPSED might impact interactions between students and teachers [17, 19, 40]. In particular, many tasks expected to be automated by AI (e.g., assessment, roll call) provide opportunities to maintain contact with students and appraise their learning journeys [38]. Relatedly, some argue that AIPSED will negatively impact students’ social development, for example due to excessive dependence on technology and reduced interaction with peers [17, 44, 52], the low emphasis placed by AIPSED on social skills [40], the individualistic outlook promoted by AIPSED [15], or the framing of learning as passive consumption rather than a process actively shaped by the students [53].

#### 4.3.3 Negative impact on teachers and educational practices

This theme deals with unwelcome implications AIPSED may have for the role and situation of the teachers, as well as the established methods of teaching. AIPSED may replace teachers [9, 17, 19, 25, 41, 44], especially when it is used as a cost-cutting measure [18](Butt et al., 2022). However, soft skills cannot be currently automated, which might protect teachers from displacement [18], and ethical use of AIPSED requires that not every task (e.g., decision making) should be fully automated [23]. LLMs cannot replace critical thinking and problem-solving skills and thus they should only support rather than substitute teachers [22]. Rowe notes that only those who understand teaching as merely transmiting information are likely lose their job, because this is the only sphere in which technology might outperform humans [35].

AIPSED will transform the role of human educators and this possibility comes with uncertainties [9, 16, 43]. AIPSED might impact teachers’ decision-making [52, 55], automation of some tasks might lead to deskilling and deprofessionalisation of teachers [38, 41] and AI might ultimately turn teachers into facilitators or supervisors of learning, rather than people managing classroom activities [15, 25, 32]. AI could gradually turn schools into software-centric organisations where human influence is slowly eroded, although ideally teachers could use AIPSED as tools to augment their skills [35]. AIPSED could increase performance-related pressures and result in stress, as the increasing use of sensors, cameras and metrics could lead to greater scrutiny of teachers’ practice [34]. Du Boulay questions to what extent teachers should respond to suggestions from AIPSED, regarding, for example, students’ engagement, and notes that how teachers divide their attention between students is an ethically salient consideration [29]. In turn, Bu writes that delegating tasks to AI impacts the student-teacher relationship, gradually placing technology in a more central position and thus reducing educators’ importance [17]. Treviranus argues that automation of routine tasks could dissuade teachers from questioning whether these tasks contribute to educational goals in any way [26].

Other concerns relate to potential changes to educational practices [39, 46]. In particular, schools are already focusing on marking and test scores and this could be further exacerbated by AI’s affinity for automatic assessment [16]. However, AI could mean that schools will move away from final exams as the technology’s ability to monitor students’ progress would make it possible to engage in continuous assessment and feedback (although the authors see this development as positive) [43]. Education might turn towards individual learning, leading students to collaborate less with teachers and peers [9]. Furthermore, it is possible that economic needs will determine which skills are emphasised by AIPSED [15], while Selwyn is concerned about the drive to encompass all learning activities within a particular digital system, especially as economic concerns would incentivise the development of scalable and context-agnostic tools (with the associated worry that such systems would not respond well to local needs) [56].

Uniformisation of education is seen as a possibility because particular AIPSED tools might deliver the same content to students from different schools, backgrounds and countries [15, 46], especially if priorities and curricula are determined in the top-down manner (e.g., at the national level [25]) or particular practices and pedagogies are scaled up [16]. Moreover, since AI performs better when dealing with standardised data, schools might be incentivised to standardise both material and assessment to facilitate using AI tools [40]. Indeed, AIPSED might supply students with homogenous content to make their learning experience easier to predict and control [17]. Treviranus argues that personalisation reduces the likelihood of learning something unexpected, and speculates that AI will likely promote already popular content [26].

This is particularly concerning, because it might be diffult to change practices once AIPSED tools are in place [38, 40]. For example, tools would likely depend on hierarchical categorisation of material and would suggest a limited range of content [26]. Moreover, since AI is trained on past data, it might entrench existing practices and reduce innovation in education [53].

Finally, authors mention that AIPSED might pose challenges to assessment by facilitating cheating or plagiarism [19, 22, 32, 33].

#### 4.3.4 Misuse and suboptimal use

In this section, we synthesise the discussion of the problems tha may arise when AIPSED is used inapprioprately due to malicious intentions or lack of relevant skills and knowledge. AIPSED systems and the associated data may be misused or used in ways that do not benefit the students [1, 19, 21, 22, 27, 32, 39, 44]. Bias and inaccuracy may arise when models are not used for their intended purposes, for example when a tool designed to quantify engagement grades participation [49]. Emotional recognition systems may be abused by teachers to punish and shame students [41]. Such types of AIPSED can help in disciplining and enforcing specific standards of behaviour, most often to the detriment of marginalised groups (e.g., neurodivergent, ethnic and racial minorities) [54]. AIPSED could also be deliberately set up to achieve goals unrelated to students’ learning and well-being. For example, collecting information about students’ home environment [24], manipulating test scores and other metrics to ensure that students progress academically and continue paying fees to their institution [53], cherrypicking data to justify (inaccurate or prejudiced) beliefs [37], deliberately excluding certain groups by refusing admission to poorer students or setting up different disciplinary measures for undesirable students [51]. AIPSED can also be abused by students who might discover strategies to game the system and achieve good test scores without studying, for example by finding text strings that are mistakenly assessed by the system as correct answers [19, 49]. As such, AIPSED might ultimately disincentivise learning [55].

AIPSED could also be used in suboptimal ways. Teachers are currently not trained to use AIPSED and might struggle to implement and reap its full benefits [5, 18, 22, 23, 30, 32, 38, 44, 46]. Teachers who do no understand the inner workings of AIPSED might be unable to determine when the system is useful or when it might be prone to inaccuracy and bias and place trust in its judgement when they should not [35].

Teachers’ beliefs and attitudes might also influence the adoption of AIPSED [31, 36, 38]. Even when AIPSED is beneficial, teachers’ scepticism about its promises might stop them from implementing it in the classroom [23] and experienced teachers might rely on practical experience rather than incorporate new technologies, in contrast to pre-service teachers who were exposed to AIPSED in their training [39]. Accordingly, some teachers might avoid using AIPSED due to their perception of risks [50, 52].

#### 4.3.5 Low transparency and interpretability

This section collects the implications connected to how the decisions and functioning of AIPSED are explained and understood to different stakeholders. Concerns over low transparency of AIPSED are prominent in the literature [29, 32, 43, 56]. Stakeholders are not provided enough information about the inner workings of AIPSED and its decisions or the data it it generates [9, 15, 18, 20, 23, 25–27, 37, 40, 41, 44, 51–53]. AIPSED might flag some students as prone to stress or likely to fail a course without disclosing contributing factors, limiting the educators’ ability to intervene [20, 23]. Yan et al. note that none of the tools they examined (*n* = 118) in their review can be considered transparent, with most being understandable only for researchers and developers [3]. Schiff argues that in some cases, the developers themselves are unable to fully explain AIPSED’s decisions [25].

This is seen as potentially alienating users [9], encouraging opposition to unexplained decisions made by AI [41], reducing trust [18, 19, 52], making it more difficult to investigate biases and limitations [49, 53], or limiting the ability of users to challenge undesirable decisions [40, 53]. However, full transparency might not always be desirable or possible. For example, Farrow argues that students and teachers require different explanations of algorithms than engineers, but adds that if AIPSED is too transparent about its teaching methods (i.e., more than human teachers), students might respond by changing how they behave and approach the material [55]. Schiff asks whether we should always want the students to know whether they are being influenced by AI, for example through nudging [25]. As such, Smuha argues that explanations of AIPSED should be adapted to stakeholders as schoolchildren have different transparency requirements than, e.g., teachers or developers [40].

Full transparency of a particular algorithm does not necessarily explain the wider AI ecosystem [55]. In some situations more transparent systems might be less effective, thus introducing a trade-off between explainability and quality of education [20]. Companies might be uninterested in disclosing how their products work to protect proprietary information [51, 53] or to avoid reputational damage connected to disclosed biases and limitations [49]. Furthermore, data, decisions and functions of AIPSED systems cannot be merely disclosed– they need to be interpreted by the involved stakeholders [9, 35, 44]. However, this process is not straightforward. The algorithms may be so complex as to become effectively “non-interpretable” ^[53(p. 317)]^ and the process in general can be considered burdensome and difficult [20]. Moreover, students and teachers may lack the ability and background knowledge necessary to engage in interpretative work [5, 23], for example because they never received relevant training, forgot the appropriate skills, or have insufficent experience [37].

Pea et al. and Wei and Niemi emphasise the need for teaching students and teachers the skills necessary to make sense of AIPSED [27, 45]. This is crucial as interpretative skills vary between individuals [40]. Disadvantaged groups might have lower digital literacy [48] or lower access to resources and advice required for interpretative work [53]. Consequently, Farrow argues that failure to attend to such differences could further the existing digital divide [55].

#### 4.3.6 Disparities in power and participation

In this section, we discuss how AIPSED affects power relations embedded in education and who has a say in the decisions surrounding the technology. AIPSED tools reflect existing power relations [9, 14, 46, 54] or exacerbate power imbalances and introduce new ones [27, 38, 42, 48, 54, 56], for example by increasing the influence of designers over the educational system [53]. Smuha observes that power disparities are inherent to schools as students, teachers and administrators all wield different amounts of influence [40]. However, the introduction of AIPSED might exacerbate these disparities (e.g., teachers may refrain from complaining about AIPSED if the administration invested significant resources into its implementation). Additionally, since developers know more about AIPSED than educators and administrators, they will wield greater control over it.

AIPSED subjects education to various economic and political pressures [48, 53] and might be influenced by, e.g., lobbying and thus reflect interests distinct from students’ and educators’ needs [18, 25]. Selwyn considers the shifts of power associated with AIPSED as an aspect of the ongoing centralisation of power within few influential platforms [56]. Furthermore, non-democratic regimes might use AIPSED for censorship [1, 45] and AIPSED might negatively impact civic and democratic education and the idea of being a good citizen [40, 48, 56].

Decisions surrounding AIPSED are not made by students, teachers or parents [14, 25] and some argue that their participation in the decision-making surrounding AIPSED is crucial for its ethical use [27, 35, 41]. This is particularly relevant in low-/middle-income countries where AIPSED will likely be implemented without consultation with local stakeholders [41].

Furthermore, teachers, are not adequately included in the design of AIPSED and thus their needs and circumstances are not reflected in available products [35, 36, 38, 54]. Arguably, some stakeholders are unable to take part in design efforts due to lack of time, infrastructure or digital literacy, which narrows down who is represented in the developed tools [35, 38, 47]. However, broad and genuine stakeholders participation is crucial for the creation of fair and equitable AIPSED [46, 49, 51] and their input is particularly important to reduce bias [15] or address privacy expectations [31, 51]. Kitto and Knight note that educators’ role in design is unclear and ask whether they should be considered participants or co-investigators [50]. Moreover, they highlight that considerations relating to dignity and autonomy require explicit input and consent from AIPSED users rather than be motivated by the findings of participatory research. Stakeholder participation might also be reduced to a box-checking exercise that ignores educators’ input in favour of developers’ decisions [54].

#### 4.3.7 Negative impact of commercial interests

This theme collects the discussion of the implications of the involvement of for-profit actors and the influence AIPSED gives them in educational settings. Integrating commercial AI tools in education allows companies to infiltrate [53] and exert control over public educational systems [23, 43, 44, 46, 53]. Companies could monopolise certain aspects of AIPSED [21, 38, 51] and thus make schools dependent on their services and infrastructure [38, 46, 48, 56] or ensure continuous profits through subscription services [43]. As such, the presence of commercial AIPSED products blurs the distinction between educational and market systems, ultimately positioning learners as consumers [15]. Similarly, private companies see schools as valuable markets and thus subject education to market logic [46].

For-profit actors might develop tools that primarily further their commercial interests rather than students’ or educators’ needs [16, 18, 23–25, 27, 38, 42, 46]. Especially, when developers do not disclose biases and limitations to avoid criticism [49], prioritise skills related to their own labour and market needs [15], inflate test scores to make the systems seem more effective [53], ignore security risks and use data for non-educational purposes [17, 21, 29], or price and bundle services in order to maximise profits [56]. Consequently, for-profit companies, and not students and educators, might ultimately benefit the most from AIPSED [46, 51, 53].

Many criticise the fact that private technology companies effectively own and control student data generated by AIPSED tools [9, 44] or note that the issue of data ownership is currently unresolved [1, 9, 23, 29, 38, 40, 44, 53]. Data commodification and monetisation are pressing issues, as authors warn against selling student data to third-parties or using them for targeted advertising [15, 17, 21, 39, 40, 45]. Such practices effectively reduce and objectify students as sources of commercially valuable information [42], but data commodification does not stop companies from charging additional fees for premium features [51]. Pinkwart concedes that monetisation of data might allow poorer users to access valuable AIPSED tools, especially as he sees advertisements as unsuitable for educational products, but argues in favor of clear rules on exchanging data for free access to services [31]. Relatedly, commodification of data affects marginalised users the most as they are more likely to cede control over their data to access vital services [53].

#### 4.3.8 Issues surrounding ethics and regulation

This section overviews the concerns connected to the limited ethical and legislative oversight over AIPSED. Ethical guidelines and regulations that adequately cover AIPSED are sorely needed [1, 9, 23, 27, 35, 38, 44, 51, 55] and two sources include calls for the development of such guidelines [28, 42]. Even where guidelines for AI exist, they lack provisions specific to education [41, 43, 47]. Baker et al. attribute this to a limited imaginary and lack of presence of AIPSED in, e.g., popular culture [15], while Schiff [41] suggests that the policy-level discussion of AIPSED prioritises economic value over ethics. Smuha notes the limitations of some regulatory efforts that focus only on cybersecurity and data protection, but argues that EU’s proposed framework of Trustworthy AI covers many concerns surrounding AIPSED [40].

Diversity of views about ethics impedes developing all-encompassing and universally agreed upon guidelines [42, 46], especially as beliefs and circumstances change over time [38]. Nemorin et al. claim that most attempts at establishing ethical guidelines for AIPSED are Western-centric and fail to incorporate other ethical positions [42]. Various regulatory regimes have different requirements [42] and since AIPSED tools might operate in multiple countries and are developed by multinational companies, it is difficult to adequately localize regulation [46]. Further, even individual regulatory schemes contain conflicting duties which further problematises enforcement [50]. The speed at which AIPSED progresses is another obstacle that leaves regulatory efforts behind [15, 46]. This is further problematised by lack of systematic studies on regulation and guidelines for AIPSED [44].

General educational guidelines, such as those aimed at teachers’ conduct, do not contain provisions regarding AIPSED [29], which means that AIPSED is often implemented into schools without ethical oversight [9]. AIPSED developers are also not bound by the same ethical standards as teachers [30], and guidelines and regulations are difficult for companies to understand and follow, often despite their best intentions [23].

Nemorin et al., also warn about conflating the existence of ethics guidelines with ethicality of AIPSED as many initiatives can be considered ethics washing and forward industry interests [42]. The lack of in-depth discussion of AIPSED ethics would cede the stage to the companies and allow them to determine what should be considered good for education [50]. Compiling ethical checklists could allow companies to escape actual oversight, while enabling them to shape guidelines and regulation [46]. Kitto & Knight [50] raise concerns about enforceability of guidelines and regulations, and Baker and Hawn argue that the proprietary nature of AIPSED tools complicate oversight [49]. This is also emphasised by Kousa & Niemi who argue that lack of enforcement increases the likelihood of companies downplaying ethical issues [23].

Lack of consideration of ethics in the literature about AIPSED is noticed in some sources [5, 9, 15, 27, 38, 45, 46]. Crompton et al., speculate that this might be due to researchers’ desire to present their work more positively [5], while Porayska-Pomsta et al. suggest that authors see their work of improving education through digital tools as inherently valuable and thus pay attention to potential risks [38]. For Selwyn, the discussion is dominated by technical formulations of ethical problems [56], while Williamson et al. suggest that authors do not label moral concerns as ethical and use other language [46]. In Du Boulay’s view, AIPSED researchers’ approach to ethics has evolved, with early work focusing on technical robustness and effectiveness, and ethics being a primary concern for current AIPSED researchers [29]. Respondents (*n* = 17) to the survey by Holmes et al. about the ethics of AIPSED agree with the lack of consideration of ethics in AIPSED and attribute it to lack of ethical expertise among AIPSED researchers [9].

#### 4.3.9 Lack of accountability

Accountability pertains to “who should be considered responsible when something goes wrong” with AIPSED ^[9(p. 508)]^. Since we are unable to determine exactly how AI decisions are made, we cannot challenge them or hold anyone accountable for them [18, 27, 40, 53].

However, other understandings of accountability are also present in the literature. Some mention accountability (or liability) as a potential concern, often without explicating what it entails [9, 38, 43]. Pea et al. question the decision-making processes behind the introduction of AIPSED in schools and position accountability as relating to whether parents and local regulation have an impact on such processes, as well as the operation of AIPSED [45]. Huang argues that it is unclear who should be held responsible for, e.g., errors in AI-powered assessment [21]. Kousa & Niemi note that it is rarely clear how to divide responsibility between the users, developers and the machine [23]. Such worries lead Li and Gu to claim that accountability is “meaningless to some degree” as humans will always end up accountable for the functioning of AI ^[52(p. 196)]^. Nguyen et al., also note that AIPSED cannot be accountable in the same manner as humans and argue that the development and regulation of AIPSED should follow responsible AI frameworks and make human stakeholders responsible for the decisions of AIPSED [44]. However, Du Boulay claims teachers will likely shoulder extra responsibilities to ensure that AI is beneficial and harmless [30]. Similar observations are found in other sources [15, 23], especially as teachers do not want to assume such responsibility and would attempt to delegate it to the companies developing AIPSED or the local education governance bodies [23]. Smuha argues that a crucial aspect in ensuring accountability lies in robust reporting mechanisms which allows stakeholders to report potential concerns and violations without fearing repercussions [40].

Two further developments complicate accountability in the context of AIPSED. First, the increasing use of AI could reduce the influence of human stakeholders on decisions surrounding education like what to teach and how [26, 35, 40, 46, 48]. As such, authors reiterate the need for human agency [9] and keeping humans in the loop [15]. Second, AIPSED tools are likely to develop in the future (often in unanticipated ways, resulting from, e.g., unsupervised learning), further complicating accountability and human oversight [18, 25, 36, 52].

## 5 Discussion

### 5.1 The ethical debate should integrate empirical and pedagogical perspectives

Many arguments found in the sample are speculative or call for more evidence. While the normative arguments are convincing and relevant, it is often unclear whether they refer to ethical implications that are likely/plausible or to ones that are directly observable. Only seven of the retrieved sources report the use of formal empirical methodologies [9, 23, 27, 41, 42, 44, 52], but many claims are substantiated through references to existing literature, policy guidelines and media discussion (and six sources report on how they identified relevant literature [1, 3, 5, 32, 52, 55]). However, it is common for ethical literature to mainly consist of conceptual work, as it is difficult to determine whether and to what extent ethical implications of AIPSED can or should be empirically observed, especially when authors discuss *potential* impacts, such as risks of harm or manipulation.

Still, future work would greatly benefit from the integration of existing and emerging empirical findings with ethical thinking. While some rightly point out that there is currently little evidence on the efficacy of AIPSED [38], there has been recent empirical work discussing, e.g., teachers’ perceptions of AI [58, 59], and some sources included in our review examine the state of the policy landscape surrounding AIPSED [41, 42, 44]. Such integration would allow for the formulation of more concrete responses to the impacts of AIPSED (as we note below, this is a significant weakness of the current literature), while also making it easier to gauge the likelihood of specific ethical impacts.

Accordingly, we recommend that future research fill this gap by conducting qualitative research exploring stakeholders’ hopes and concerns surrounding AIPSED (e.g., through focus groups, and DELPHI studies) and the challenges and opportunities emerging through the use of AIPSED (e.g., through interviews and ethnographic observation), as well as large scale quantitative surveys comparing the ethical implications of AIPSED across different countries and stakeholder groups. An interesting example of such work is the study by Swist et al. which designed a game that helped students explore the controversies surrounding algorithm-assisted grading [60]. In turn, Rahm asked teacher students to develop fictional narratives discussing evil AIPSED tools and reflect upon the problems they would introduce in the classroom [61].

Similar research would help contextualise and develop the claims about the pedagogy of AIPSED. While many authors criticised the lack of pedagogical thinking underlying AIPSED or raised concerns about how the teachers’ role will be transformed by AI, they neither discussed what appropriate pedagogies would entail nor suggested how teachers should respond to the impact of AIPSED (with the notable exception of Rowe who discussed which skills should be embraced by the teachers [35]). As such, we suggest that education scholars and philosophers of education should focus on developing new approaches to teaching and learning alongside AI, as well as lists of best practices that would help educators navigate the technologically-augmented landscape. While new relevant publications have come out since we started work on this review [62–64], further work on AIPSED pedagogies is warranted.

### 5.2 The literature does not offer clear and actionable recommendations

The literature identifies ethical implications but authors rarely engage in evaluation, analysis, and formulation of substantive recommendations. Much of the literature focuses on asking (critical) questions or presenting benefits and concerns, but there is little discussion of value conflicts, and authors generally do not take normative positions in the debate that could point to concrete responses to the discussed implications (with some exceptions, e.g., specific pathways to alleviate bias and inequalities discussed by Holstein & Doroudi [36]). Even harsh criticism is not accompanied by substantive recommendations. For example, while Madaio et al., provide an in-depth analysis of the oppressive nature of AIPSED rooted in contemporary structures of discrimination (along racist, ableist and economic lines), their conclusion merely calls for greater inclusivity in design and greater variety of theories used to forward work on AIPSED (e.g., queer theories) [54]. Similarly, although Selwyn is highly critical of the environmental impact of AIPSED, he merely posits that the use of such technologies “makes little sense” as a result ^[56(p. 627)]^.

The enumeration of pros and cons forms a significant part of the ethical studies of new technologies. However, an in-depth ethical analysis entails the balancing of competing values and a discussion of involved trade-offs. This should be detailed and conclusive enough to enable the formulation of substantive recommendation that would allow stakeholders to alleviate the ethical impacts of the technology and harness its benefits. The scarcity of recommendations is particularly concerning as authors attribute the impacts discussed in this review to inherent features of AIPSED and structural injustices embedded in educational systems rather than simple errors. As such, it is unlikely that they will be addressed without coordinated efforts on the part of regulators, designers and educators.

We see two potential reasons why authors often only identify ethical implications and do not formulate recommendations. The computational focus of many of the papers may influence the debate, which is particularly evident in the discussion of bias where the impacts are presented as remediable through improved design and greater representation in data rather than wide reaching social changes or regulatory interventions. However, as authors primarily adopt technical and social scientific perspectives, we speculate that they may be reluctant to adopt normative positions or advocate for solutions associated with such positions. As such, we believe that the current debate would greatly benefit from the involvement of philosophers, particularly those working in applied ethics who are involved in moral debates and are used to assessing value conflicts or formulating specific prescriptions to avoid a moral impasse. None of the sources included in this review were published in philosophical journals and even though philosophers have joined the debates on other types of AI, they have been slow to consider AIPSED. Similarly, the involvement of legal scholars would further the work on regulatory recommendations and would help determine which legal measures should be taken to ensure the beneficence of AIPSED.

### 5.3 Engaging a wider variety of voices

It is worth noting that our sample predominantly looked at AIPSED from a birds-eye view or from the teachers’ perspective. Although some authors examined what problems might arise from the point of view of the students (such as the inability to maintain privacy from their parents) [31, 47], there was no empirical paper in our sample that actually involved students and their parents as participants (even though some sources contained excerpts from interviews and surveys with teachers or researchers/developers [9, 23]). We already noted above that ethical reflection on AIPSED should be coupled with participatory research, but it is worth highlighting some difficulties inherenet to attempts at greater integration of students’ and parents’ perspective. Primary and secondary school students are (predominantly) minors, which complicates their participation in empirical studies as their vulnerable status often (and justifiably) requires researchers to provide additional accommodations and obtain additional permissions (e.g., from a research ethics committee). This might discourage some from seeking students’ input, especially as the use of AI in research (e.g., walkthrough interviews reporting on students’ use of AIPSED) would warrant additional caution and scrutiny. In turn, while parents should be involved in their childrens’ education and the decisions surrounding it, they already face many competing demands for their time and attention, so it is not surprising that they might not prioritise research participation or that researchers might direct their efforts elsewhere. We are also mindful of the fact that while stakeholder involvement is fundamental for a complete understanding of the impacts of AIPSED, their perspective might be limited. For example, it is doubtful whether children would offer genuine insights on the ethics of AIPSED, especially since many efforts to promote AI literacy are still in their infancy.

Our review also partially supports claims that the majority of research on the ethics of AIPSED is undertaken in Western countries [6]. However, it also highlights several interesting papers written or co-written by scholars based in Asia [17, 21, 27] and we suspect that their number would have been higher had we included languages other than English. It should be noted that many sources written by non-Western scholars can be found in different, often less prestigious, publications than the work by their Western counterparts. As such, work from different communities rarely intersects, which leads us to call for wider international collaboration on the ethics of AIPSED. This is particularly important as our review highlights the varied potential for uptake and varied impact of AIPSED between countries. In this context, we suggest that cross-country comparative studies might serve a dual purpose. On the one hand, it would be valuable to learn how stakeholders’ hopes and concerns differ across countries or which issues are going to have the greatest impact in particular regions. On the other, the need to incorporate various perspectives and local expertise in such studies would promote collaboration across research communities which, as our review indicates, currently do not intersect. Non-Western philosophical framework might also offer fruitful tools for the analysis of the ethical implications of AIPSED. For example, Reviglio & Alunge recently highlighted that Ubuntu philosophy offers an interesting counterpoint to the discussions of privacy by diverging from the Western-centric discussions of privacy as an individual right [65]. Similarly, ethical analyses conducted from a Confucian or Buddhist standpoint might challenge the individualist slant of much of Western philosophy– which might be especially relevant for a highly communal context, such as education.

Finally, we need to highlight that the discussions surrounding the ethics of AIPSED are ongoing and accelerating, and no review can capture their entire scope. For example, we already noted that the more technical AIED conferences have recently started encouraging ethics-focused discussions and there has been great interest in AIPSED within media and at the policy level. Consequently, we want to emphasise the need to broaden the scope of the debate on the ethics of AIPSED both within and outside of the academic circles as the breadth of relevant and worthwhile arguments cannot be captured by any individual review.

## 6 Conclusion

This paper presented the results of a systematic review on the ethical implications of AIPSED. Our search identified 48 relevant sources and thematic analysis of the arguments presented within them helped us outline 17 themes. Four of these dealt with the positive impacts of AI on teachers’ jobs, reduction of educational inequalities, improvement of educational outcomes and a range of other benefits that did not fit neatly into an overarching category. In turn, 13 themes included negative impacts: privacy risks, unfairness and inaccuracy of models, amplification of educational injustices, harms (including to autonomy), the limitations of technology, its negative impact on learning, as well as educational practices and teachers’ roles, misuse of the technology, its low transparency and interpretability challenges, disparities in ability to shape the development and deployment of AIPSED, the growing influence of private companies on education, limited discussion of ethics and regulation, and questions surrounding accountability.

While the analyses included in our review do not allow for substantive recommendations, we still think it worthwhile to outline some sketch ideas for alleviating the negative impacts of AIPSED and we suggest here three related recommendations. First, many concerns listed by the authors stem from the profit-driven nature of many AIPSED tools (e.g., those related to privacy, influence of private companies, or even manipulation). For this reason, educational policymakers would do well to extend greater oversight over the implementation of AI in schools. Of course, the regulatory landscape is evolving and following early initiatives such as the Beijing Consensus on Artificial Intelligence in Education, more bodies are joining regulatory efforts– in particular, the EU AI Act bans emotion recognition in schools. However, it might be worthwhile for some countries and international organisations (e.g., the EU) to consider the public development of AIPSED as an alternative to privately-suppled tools.

Second, we suggest that the adoption of AIPSED should be based on robust procurement processes rather than depend on the availability and novelty of technology. The discussions on the misguided pedagogical assumptions of AIPSED, their mismatch for educational purposes and their inherent limiations highlight that new tools are not always deployed to address the needs of teachers and students. Consequently, rather than allowing companies the shape the narratives surrounding AIPSED, it is educational policymakers who should determine which purposes and values ought to be served by educational technology. Clearly defined procurement terms would allow schools to order the development of tools that fit their requirements instead of merely choosing from a range of available options.

Third, since much of the discussion surrounding the ethics of AIPSED is based on speculation or even hype, we argue that policy efforts should be guided through input from stakeholders and empirical evidence. Consequently, we highlighted in the discussion the kind of research that would greatly enhance decision-making in the AIPSED space, as well as the need to include a wider variety of voices in the debates on the ethical implications of the technology.

This review, overall, highlights that the ethical implications of AIPSED are not clear-cut. Despite some widely discussed positive developments, there are many challenges that need to be addressed if we want to ensure that the technology benefits the students and teachers alike. We hope that our review offers researchers clear directions for work that would further our understanding of the ethics of educational AI, while also presenting policymakers with an in-depth and comprehensive analysis of the competing ethical considerations that need to be considered in the adoption and regulation of the technology.

## Supplementary Material

Supplementary File 1

Supplementary File 2

**Supplementary Information** The online version contains supplementary material available at https://doi.org/10.1007/s43681-025-00770-0.

## Figures and Tables

**Table 1:** Overview of results

Themes	Number of sources

Benefits of AIPSED	

Facilitating teachers’ jobs	25
Reducing inequalities	23
Improving educational outcomes	23
Other benefits	10
**Concerns about AIPSED**	
Privacy challenges	41
Unfairness and low accuracy	41
Perpetuating injustice	39
Negative impact on autonomy and other harms	35
Limitations of technology	31
Negative impact on learning	30
Negative impact on teachers and educational practices	26
Misuse and suboptimal use of AIPSED	27
Low transparency and interpretability	26
Disparities in power and participation	24
Negative impact of commercial interests	26
Issues surrounding ethics and regulation	23
Lack of accountability	20

## Data Availability

No datasets were generated or analysed during the current study.

## Footnotes

1 As quoted by Holmes and Tuomi ^[7(p. 546)]^.

2 This is also a practical consideration as the number of papers to be included in the review could become impossible to manage without a narrower focus.

3 We considered the inclusion of conference materials, but decided against doing so as the publication of conference proceedings is not very common in humanities and social science– areas which we expected to supply the majority of the literature. At the same time, until very recently key technical AIED conferences did not include dedicated ethics discussions and papers mainly focused on the ethics of doing AIED research, rather than on the ethical implications of AIED [9]. However, we note in the discussion that this decision narrows down the scope of our findings.
