# Supplementary material for: Unpacking the ethics of using AI in primary and secondary education: a systematic literature review
Source: AI Ethics. Author manuscript; Available in PMC 2025 Sep 16. (PMC12434897; doi:10.1007/s43681-025-00770-0)
Supplement: Supplementary File 1 [file NIHMS2106841-supplement-Supplementary_File_1.docx]

**Unpacking the Ethics of Using AI in Primary and Secondary Education:**

**A Systematic Literature Review**

**Supplementary File 1 – elaboration of search terms and search strings**

The first set of search terms used in th review contains terms commonly used to discuss the use of AI in education and consists of the acronym “AIED” and phrases “artificial intelligence in education”, “AI in education”, “AI in school*”, “artificial intelligence in school*”, “AI in K-12”, “artificial intelligence in K-12”, “AI for education” and “artificial intelligence in education”. We deliberately chose broad terms (i.e., did not confine our search to only cover AI in primary and secondary education) to capture as many relevant results as possible and manually excluded irrelevant sources (e.g., those discussing AI only in the context of higher education). We considered splitting this set of terms into two (i.e., one set containing terms relating to AI and another with terms relating to education), but this resulted in thousands of irrelevant search results (e.g., discussing the teaching of AI literacy in medical schools or the use of AI to track schools of fish). Similarly, we decided not to include terms such as teaching and learning as they returned too many irrelevant results, e.g., ML in general.

The second set was added to further refine the results by only retrieving sources discussing the ethics of adopting AIPSED. We do not confine ethics only to the negative aspects of AIPSED, but seek to provide an account of all aspects of its use that the authors consider normatively relevant. Moreover, since ethics often stretches beyond the domain of philosophy, especially in the context of the AI ethics debate, researchers from different disciplines (e.g., computer science or education studies) might use non-philosophical terminology to engage in normative discussions. Consequently, we included a variety of terms such as: ethic*, moral*, virtue*, norm*, value*, risk*, concern*, challenge*, opportunit*, promis*. In our exploratory search, these terms enabled us to extract the highest number of relevant sources. We adapted both of the sets into search strings as required by the used databases (Scopus, Web of Science, Academic Search Complete and Google Scholar). Exact search strings:

1.     Scopus

TITLE-ABS-KEY ( "aied" OR "artificial intelligence in education" OR "ai in education" OR "artificial intelligence in school*" OR "ai in school*" OR "artificial intelligence in k-12" OR "ai in k-12" OR "ai for education" OR "artificial intelligence for education" ) AND TITLE-ABS-KEY ( ethic* OR moral* OR virtue* OR norm* OR value* OR risk* OR concern* OR challenge* OR opportunit* OR promis*) AND ( LIMIT-TO ( DOCTYPE,"ar" ) OR LIMIT-TO ( DOCTYPE,"ch" ) OR LIMIT-TO ( DOCTYPE,"bk" ) ) AND ( LIMIT-TO ( LANGUAGE,"English" ) )

2.     Web of Science:

(TI=(( "aied" OR "artificial intelligence in education" OR "ai in education" OR "artificial intelligence in school*" OR "ai in school*" OR "artificial intelligence in k-12" OR "ai in k-12" OR "ai for education" OR "artificial intelligence for education" ) AND ( ethic* OR moral* OR virtue* OR norm* OR value* OR risk* OR concern* OR challenge* OR opportunit* OR promis*)) OR AB=(( "aied" OR "artificial intelligence in education" OR "ai in education" OR "artificial intelligence in school*" OR "ai in school*" OR "artificial intelligence in k-12" OR "ai in k-12" OR "ai for education" OR "artificial intelligence for education" ) AND ( ethic* OR moral* OR virtue* OR norm* OR value* OR risk* OR concern* OR challenge* OR opportunit* OR promis*)) OR AK=(( "aied" OR "artificial intelligence in education" OR "ai in education" OR "artificial intelligence in school*" OR "ai in school*" OR "artificial intelligence in k-12" OR "ai in k-12" OR "ai for education" OR "artificial intelligence for education" ) AND ( ethic* OR moral* OR virtue* OR norm* OR value* OR risk* OR concern* OR challenge* OR opportunit* OR promis*))) AND (DT==("ARTICLE" OR "BOOK CHAPTER"))

3.     Academic Search Complete:

TI ( ( "aied" OR "artificial intelligence in education" OR "ai in education" OR "artificial intelligence in school*" OR "ai in school*" OR "artificial intelligence in k-12" OR "ai in k-12" OR "ai for education" OR "artificial intelligence for education" ) AND ( ethic* OR moral* OR virtue* OR norm* OR value* OR risk* OR concern* OR challenge* OR opportunit* OR promis*) ) OR AB ( ( "aied" OR "artificial intelligence in education" OR "ai in education" OR "artificial intelligence in school*" OR "ai in school*" OR "artificial intelligence in k-12" OR "ai in k-12" OR "ai for education" OR "artificial intelligence for education" ) AND ( ethic* OR moral* OR virtue* OR norm* OR value* OR risk* OR concern* OR challenge* OR opportunit* OR promis*) )

OR KW ( ( "aied" OR "artificial intelligence in education" OR "ai in education" OR "artificial intelligence in school*" OR "ai in school*" OR "artificial intelligence in k-12" OR "ai in k-12" OR "ai for education" OR "artificial intelligence for education" ) AND ( ethic* OR moral* OR virtue* OR norm* OR value* OR risk* OR concern* OR challenge* OR opportunit* OR promis*) )

4.     Google Scholar:

allintitle: "aied" OR "artificial intelligence in education" OR "ai in education" OR "ai for education" OR "artificial intelligence for education" AND ethics OR ethical OR moral OR virtues OR virtue OR norms OR normative OR values OR challenges OR concerns OR risks OR opportunities OR promises
