# Supplementary material for: Unpacking the ethics of using AI in primary and secondary education: a systematic literature review
Source: AI Ethics. Author manuscript; Available in PMC 2025 Sep 16. (PMC12434897; doi:10.1007/s43681-025-00770-0)
Supplement: Supplementary File 2 [file NIHMS2106841-supplement-Supplementary_File_2.docx]

**Unpacking the Ethics of Using AI in Primary and Secondary Education:**

**A Systematic Literature Review**

**Supplementary File 2. Sources included in the review**

Akgun, S., & Greenhow, C. (2022). Artificial Intelligence (AI) in Education: Addressing Ethical Challenges in K-12 Settings. *AI and Ethics*, *2*, 431–440. <https://doi.org/10.1007/s43681-021-00096-7>

Alshahrani, A. (2023). The impact of ChatGPT on blended learning: Current trends and future research directions. In *International Journal of Data and Network Science* (Vol. 7, Issue 4, pp. 2029–2040). <https://doi.org/10.5267/j.ijdns.2023.6.010>

Baker, B., Mills, K. A., McDonald, P., & Wang, L. (2023). AI, Concepts of Intelligence, and Chatbots: The ‘Figure of Man,’ the Rise of Emotion, and Future Visions of Education. *Teachers College Record*, *125*(6), 60–84. <https://doi.org/10.1177/01614681231191291>

Baker, R. S., & Hawn, A. (2022). Algorithmic Bias in Education. In *International Journal of Artificial Intelligence in Education* (Vol. 32, Issue 4, pp. 1052–1092). <https://doi.org/10.1007/s40593-021-00285-9>

Bartoletti, I. (2022). AI in education: An opportunity riddled with challenges. In W. Holmes & K. Porayska-Pomsta (Eds.), *The Ethics of Artificial Intelligence in Education: Practices, Challenges, and Debates* (pp. 74–90). Routledge. <https://doi.org/10.4324/9780429329067-5>

Berendt, B., Littlejohn, A., & Blakemore, M. (2020). AI in education: Learner choice and fundamental rights. In *Learning, Media and Technology* (Vol. 45, Issue 3, pp. 312–324). <https://doi.org/10.1080/17439884.2020.1786399>

Bu, Q. (2022). Ethical Risks in Integrating Artificial Intelligence into Education and Potential Countermeasures. *Science Insights*, *41*(1), 561–566. <https://doi.org/10.15354/si.22.re067>

Butt, U. J., Davelis, A., Abbod, M., Eghan, C., & Agbo, H.-M. (2022). Improving Learning Experience and Privacy in Education Using the Power of Big Data and Artificial Intelligence. In S. Singh Dadwal, H. Jahankhani, & A. Hassan (Eds.), *Integrated Business Models in the Digital Age Principles and Practices of Technology Empowered Strategies* (pp. 371–424). Palgrave Macmillan. <https://doi.org/10.1007/978-3-030-97877-8_11>

Corbeil, M. E., & Corbeil, J. R. (2021). Establishing Trust in Artificial Intelligence in Education. In J. Paliszkiewicz & K. Chen (Eds.), *Trust, Organizations and the Digital Economy: Theory and Practice* (pp. 49–60). Routledge. <https://doi.org/10.4324/9781003165965-5>

Crompton, H., Jones, M. V., & Burke, D. (2022). Affordances and challenges of artificial intelligence in K-12 education: A systematic review. In *Journal of Research on Technology in Education*. <https://doi.org/10.1080/15391523.2022.2121344>

Dieterle, E., Dede, C., & Walker, M. (2022). The cyclical ethical effects of using artificial intelligence in education. In *AI and Society*. <https://doi.org/10.1007/s00146-022-01497-w>

Dignum, V. (2021). The role and challenges of education for responsible ai. In *London Review of Education* (Vol. 19, Issue 1, pp. 1–11). <https://doi.org/10.14324/LRE.19.1.01>

Du Boulay, B. (2022a). Artificial Intelligence in Education and Ethics. In O. Zawacki-Richter & I. Jung (Eds.), *Handbook of Open, Distance and Digital Education* (pp. 93–108). Springer. <https://doi.org/10.1007/978-981-19-2080-6_6>

Du Boulay, B. (2022b). The overlapping ethical imperatives of human teachers and their Artificially Intelligent assistants. In W. Holmes & K. Porayska-Pomsta (Eds.), *The Ethics of Artificial Intelligence in Education*. Routledge.

Farrow, R. (2023). The possibilities and limits of XAI in education: A socio-technical perspective. In *Learning, Media and Technology* (Vol. 48, Issue 2, pp. 266–279). <https://doi.org/10.1080/17439884.2023.2185630>

Filgueiras, F. (2023). Artificial intelligence and education governance. In *Education, Citizenship and Social Justice*. <https://doi.org/10.1177/17461979231160674>

Gillani, N., Eynon, R., Chiabaut, C., & Finkel, K. (2023). Unpacking the “Black Box” of AI in Education. In *Educational Technology and Society* (Vol. 26, Issue 1, pp. 99–111). <https://doi.org/10.30191/ETS.202301_26(1).0008>

Holmes, W., Porayska-Pomsta, K., Holstein, K., Sutherland, E., Baker, T., Shum, S. B., Santos, O. C., Rodrigo, M. T., Cukurova, M., Bittencourt, I. I., & Koedinger, K. R. (2022). Ethics of AI in Education: Towards a Community-Wide Framework. In *International Journal of Artificial Intelligence in Education* (Vol. 32, Issue 3, pp. 504–526). <https://doi.org/10.1007/s40593-021-00239-1>

Holstein, K., & Doroudi, S. (2022). Equity and Artificial Intelligence in education. In W. Holmes & K. Porayska-Pomsta (Eds.), *The Ethics of Artificial Intelligence in Education* (pp. 152–173). Routledge.

Huang, L. (2023). Ethics of artificial intelligence in education: Student privacy and data protection. *Science Insights Education Frontiers*, *16*(2), 2577–2587. <https://doi.org/10.15354/sief.23.re202>

Kasneci, E., Sessler, K., Küchemann, S., Bannert, M., Dementieva, D., Fischer, F., Gasser, U., Groh, G., Günnemann, S., Hüllermeier, E., Krusche, S., Kutyniok, G., Michaeli, T., Nerdel, C., Pfeffer, J., Poquet, O., Sailer, M., Schmidt, A., Seidel, T., & Stadler, M. (2023). ChatGPT for good? On opportunities and challenges of large language models for education. *Learning & Individual Differences*, *103*. <https://doi.org/10.1016/j.lindif.2023.102274>

Kitto, K., & Knight, S. (2019). Practical ethics for building learning analytics. In *British Journal of Educational Technology* (Vol. 50, Issue 6, pp. 2855–2870). <https://doi.org/10.1111/bjet.12868>

Kousa, P., & Niemi, H. (2023). Artificial Intelligence Ethics from the Perspective of Educational Technology Companies and Schools. In H. Niemi, R. D. Pea, & Y. Lu (Eds.), *AI in Learning: Designing the Future* (pp. 283–296). Springer International Publishing. <https://doi.org/10.1007/978-3-031-09687-7>

Lameras, P., & Arnab, S. (2022). Power to the Teachers: An Exploratory Review on Artificial Intelligence in Education. In *Information (Switzerland)* (Vol. 13, Issue 1). <https://doi.org/10.3390/info13010014>

Leaton Gray, S. (2020). Artificial intelligence in schools: Towards a democratic future. In *London Review of Education* (Vol. 18, Issue 2, pp. 163–177). <https://doi.org/10.14324/LRE.18.2.02>

Li, S., & Gu, X. (2023). A Risk Framework for Human-centered Artificial Intelligence in Education: Based on Literature Review and Delphi–AHP Method. In *Educational Technology and Society* (Vol. 26, Issue 1, pp. 187–202). <https://doi.org/10.30191/ETS.202301_26(1).0014>

Luan, H., Geczy, P., Lai, H., Gobert, J., Yang, S. J., Ogata, H., Baltes, J., Guerra, R., Li, P., & Tsai, C.-C. (2020). Challenges and future directions of big data and artificial intelligence in education. *Frontiers in Psychology*, *11*, 580820.

Madaio, M., Blodgett, S. L., Mayfield, E., & Dixon-Román, E. (2022). Beyond “fairness”: Structural (in)justice lenses on AI for education. In W. Holmes & K. Porayska-Pomsta (Eds.), *The Ethics of Artificial Intelligence in Education*. Routledge.

Mavrikis, M., Cukurova, M., Di Mitri, D., Schneider, J., & Drachsler, H. (2021). A short history, emerging challenges and co-operation structures for Artificial Intelligence in education. *Bildung Und Erziehung*, *74*(3), 249–263.

Mohammed, P. S., & Watson, E. (2019). Towards inclusive education in the age of artificial intelligence: Perspectives, challenges, and opportunities. In J. Knox, Y. Wang, & M. Gallagher (Eds.), *Artificial Intelligence and Inclusive Education: Speculative futures and emerging practices* (pp. 17–37). Springer.

Nemorin, S., Vlachidis, A., Ayerakwa, H. M., & Andriotis, P. (2023). AI hyped? A horizon scan of discourse on artificial intelligence in education (AIED) and development. In *Learning, Media and Technology* (Vol. 48, Issue 1, pp. 38–51). <https://doi.org/10.1080/17439884.2022.2095568>

Nguyen, A., Ngo, H. N., Hong, Y., Dang, B., & Nguyen, B.-P. T. (2023). Ethical principles for artificial intelligence in education. In *Education and Information Technologies* (Vol. 28, Issue 4, pp. 4221–4241). <https://doi.org/10.1007/s10639-022-11316-w>

Pea, R. D., Biernacki, P., Bigman, M., Boles, K., Coelho, R., Docherty, V., Garcia, J., Lin, V., Nguyen, J., Pimentel, D., Pozos, R., Reynante, B., Roy, E., Southerton, E., Suzara, M., & Vishwanath, A. (2023). Four Surveillance Technologies Creating Challenges for Education. In H. Niemi, R. D. Pea, & Y. Lu (Eds.), *AI in Learning: Designing the Future* (pp. 317–329). Springer International Publishing. <https://doi.org/10.1007/978-3-031-09687-7>

Pinkwart, N. (2016). Another 25 Years of AIED? Challenges and Opportunities for Intelligent Educational Technologies of the Future. In *International Journal of Artificial Intelligence in Education* (Vol. 26, Issue 2, pp. 771–783). <https://doi.org/10.1007/s40593-016-0099-7>

Porayska-Pomsta, K., Holmes, W., & Nemorin, S. (2023). The ethics of AI in education. In B. DuBoulay, A. Mitrovic, & K. Yacef (Eds.), *Handbook of Artificial Intelligence in Education* (pp. 571–604). Edward Elgar Publishing Ltd.

Reiss, M. J. (2021). The use of AI in education: Practicalities and ethical considerations. In *London Review of Education* (Vol. 19, Issue 1, pp. 1–14). <https://doi.org/10.14324/LRE.19.1.05>

Rowe, M. (2019). Shaping Our Algorithms Before They Shape Us. In J. Knox, Y. Wang, & M. Gallagher (Eds.), *Artificial Intelligence and Inclusive Education: Speculative Futures and Emerging Practices* (pp. 151–163). Springer. <https://doi.org/10.1007/978-981-13-8161-4_9>

Saputra, I., Astuti, M., Sayuti, M., & Kusumastuti, D. (2023). Integration of Artificial Intelligence in Education: Opportunities, Challenges, Threats and Obstacles. A Literature Review. *Indonesian Journal of Computer Science*, *12*(4).

Schiff, D. (2021). Out of the laboratory and into the classroom: The future of artificial intelligence in education. In *AI and Society* (Vol. 36, Issue 1, pp. 331–348). <https://doi.org/10.1007/s00146-020-01033-8>

Schiff, D. (2022). Education for AI, not AI for Education: The Role of Education and Ethics in National AI Policy Strategies. In *International Journal of Artificial Intelligence in Education* (Vol. 32, Issue 3, pp. 527–563). <https://doi.org/10.1007/s40593-021-00270-2>

Selwyn, N. (2022). The future of AI and education: Some cautionary notes. In *European Journal of Education* (Vol. 57, Issue 4, pp. 620–631). <https://doi.org/10.1111/ejed.12532>

Smuha, N. A. (2022). Pitfalls and pathways for Trustworthy Artificial Intelligence in education. In W. Holmes & K. Porayska-Pomsta (Eds.), *The Ethics of Artificial Intelligence in Education*. Routledge.

Su, J., & Yang, W. (2023). Unlocking the Power of ChatGPT: A Framework for Applying Generative AI in Education. In *ECNU Review of Education* (Vol. 6, Issue 3, pp. 355–366). <https://doi.org/10.1177/20965311231168423>

Sun, F., & Ye, R. (2023). Moral Considerations of Artificial Intelligence. In *Science and Education* (Vol. 32, Issue 1, pp. 1–17). <https://doi.org/10.1007/s11191-021-00282-3>

Treviranus, J. (2022). Learning to learn differently. In W. Holmes & K. Porayska-Pomsta (Eds.), *The Ethics of Artificial Intelligence in Education*. Routledge.

Wei, G., & Niemi, H. (2023). Ethical Guidelines for Artificial Intelligence-Based Learning: A Transnational Study Between China and Finland. In H. Niemi, R. D. Pea, & Y. Lu (Eds.), *AI in Learning: Designing the Future* (pp. 265–282). Springer International Publishing. <https://doi.org/10.1007/978-3-031-09687-7>

Williamson, B., Eynon, R., Knox, J., & Davies, H. (2023). Critical perspectives on AI in education: Political economy, discrimination, commercialization, governance and ethics. In B. DuBoulay, A. Mitrovic, & K. Yacef (Eds.), *Handbook of Artificial Intelligence in Education* (Vol. 5, pp. 553–570). Edward Elgar Publishing Ltd.

Yan, L., Sha, L., Zhao, L., Li, Y., Martinez‐Maldonado, R., Chen, G., Li, X., Jin, Y., & Gašević, D. (2023). Practical and ethical challenges of large language models in education: A systematic scoping review. *British Journal of Educational Technology*, 1.\
